# Genetic and pathogenic characterizations of a naturally occurring reassortant and homologous recombinant strain of the classical infectious bursal disease virus re-emerging in chickens in southern China

**DOI:** 10.3389/fmicb.2023.1293072

**Published:** 2023-11-21

**Authors:** Weiwei Wang, Jun Shi, Yan Zhang, Yuanzheng Qiao, Wenbo Zuo, Zhiyuan Wang, Tingbin Nong, Shichen Hu, Yang Chen, Ruiyang Kong, Xiumiao He, Ping Wei

**Affiliations:** ^1^Institute for Poultry Science and Health, Guangxi University, Nanning, China; ^2^Guangxi Key Laboratory for Polysaccharide Materials and Modifications, School of Marine Sciences and Biotechnology, Guangxi Minzu University, Nanning, China

**Keywords:** infectious bursal disease virus, classical IBDV, full-length genome, reassortment, recombination, cell tropism, pathogenicity

## Abstract

Infectious bursal disease (IBD) classical virus strain (cIBDV) can cause morbidity and mortality in young chickens with severe long-term immunosuppression. However, since the emergence and widespread prevalence of very virulent strain (vvIBDV) in China from 1991, reports of cIBDV have become rare. A novel reassortant and recombinant strain GXYL211225 (genotype A1aB1a) with segment A originating from the classical strain (A1a) and segment B from the attenuated vaccine strain (B1a) was characterized in the study. Notably, segment A resulted from recombination between the cIBDV strains 150127-0.2 and Faragher52-70, expressing as a backbone from 150127-0.2, where a fragment located at the position of nucleotide (nt) 519-1 410 was replaced by the corresponding region of Faragher52-70. The infection of GXYL211225 caused mortality in SPF chicken embryos, despite lacking the critical amino acid (aa) residues 253H, 279 N and 284A associated with the cellular tropism, and induced significant cytopathic effect (CPE) on a wide range of cells, confirming its natural cell-adapted character. Furthermore, the challenge experiment of GXYL211225 was performed on the commercial Three-yellow chickens of 4-week-old, and with the vvIBDV HLJ-0504-like strain NN1172 and the novel variant (nv) IBDV strain QZ191002 as the comparison. All the challenged birds experienced reduced body-weight gain. QZ191002 infected birds showed no obvious clinical symptoms or mortality, while those of NN1172 and GXYL211225 showed typical IBD symptoms and resulted in 20% (2/10) and 10% (1/10) of mortality rates, respectively. At 7 days post-challenge (dpc), the damages of bursal of Fabricius (BF) varied among groups, with NN1172 causing the most severe lesions, followed by GXYL211225, and then QZ191002. It was also found that the pathogenicity was correlated positively with the viral load, aligning with the histopathological severity in BF. The study confirms the rapid and diverse evolution of the re-emerged classical strains in the field and emphasizes the need to monitor the changes of IBDV on both the genetic and pathogenic aspects for the effective control of the disease.

## Highlights

- A naturally occurring reassortant and recombinant classical strain GXYL211225 (genotype A1aB1a) was isolated and characterized for the first time.
- Segment A of GXYL211225 is a result of recombination between the classical strains 150127-0.2 (major parent) and Faragher52-70 (minor parent), and with segment B reasserting with the attenuated vaccine strain.
- GXYL211225 exhibits an extensive cell-tropism and has been confirmed to be a naturally occurring cell-adapted strain.
- The virulence of GXYL211225 was intermediate between that of the vvIBDV NN1172 and nvIBDV QZ191002, causing a 10% mortality rate and severe immune organ damages in chickens.

## 1 Introduction

Infectious bursal disease (IBD) is an acute, highly contagious, immunosuppressive poultry disease caused by infectious bursal disease virus (IBDV) (Muller et al., 2003; Alkie and Rautenschlein, 2016). IBDV mainly damages the avian central immune organ, the bursa of Fabricius (BF), and causes varying degrees of lesions in other immune organs (Sharma et al., 2000; Dulwich et al., 2018). IBDV belongs to the *Birnaviridae* family of *Avibirnavirus* and is a non-enveloped icosahedral virus composed of two segments (segment A and segment B) of double-stranded RNA (Dobos et al., 1979; Muller et al., 1979). Segment A contains two partially overlapping open reading frames (ORFs), where the small ORF encodes the VP5 protein, and the large ORF encodes the polyproteins VP2-VP4-VP3, which is processed into VP2, VP4, and VP3 through autolytic cleavage (Birghan et al., 2000; Coulibaly et al., 2005). VP2 serves as a major structural protein and a host-protective immunogen that is closely related to IBDV cell tropism, antigenic variation and virulence (Brandt et al., 2001; Letzel et al., 2007; Jackwood et al., 2008; Alfonso-Morales et al., 2013). Segment B encodes only the VP1 protein possessing RNA-dependent RNA polymerase activity, which plays an important role in viral genome replication, translation and virulence (Boot et al., 2005; Wang W. et al., 2021).

Currently, IBDV is classified into two serotypes (I and II), but only serotype I has been confirmed to be pathogenic in chickens (Jackwood et al., 1982; Ismail et al., 1988). Traditionally, serotype I IBDV can be further divided into classical strain (cIBDV), very virulent strain (vvIBDV), antigenic variant IBDV (avIBDV), and attenuated vaccine strain (attIBDV) (Rautenschlein et al., 2003; Jackwood et al., 2018; Islam et al., 2021; Wang et al., 2022b). The cIBDV in China was first reported in 1979, causing huge economic losses in the poultry industry at that time (Zhou et al., 1982). Since the emergence and widespread prevalence of vvIBDV in 1991, reports of classical strains in China have become rare (Zhang et al., 2022). In 2016, novel variant strain (nvIBDV) emerged in multiple regions of China, causing subclinical symptoms in chicken flocks without apparent morbidity and mortality (Fan et al., 2019; Xu et al., 2020; Hou et al., 2022; Huang et al., 2022; Wang et al., 2022a,b). Notably, since 2020, cIBDV infections have been re-emerged in several poultry-producing provinces in southern China, with their prevalence increasing year by year (Author unpublished data). Up to now, China’s field situations demonstrate the co-circulation of various strains including vvIBDV, nvIBDV and cIBDV, posing new challenges to the diagnosis, prevention and control of IBD in clinical practice.

The evolution of IBDV (segmented dsRNA viruses) primarily involves gene mutations, segment reassortment and intra-segment homologous recombination, which play a crucial role in the emergence and dissemination of the novel variant/reassortant/recombinant strains (He et al., 2009, 2014, 2016). The co-circulation of different types of strains in the field and the widespread use of live attenuated vaccines have increased the potential for reassortment /homologous recombination among different IBDV strains (He et al., 2009; Wang et al., 2022b). Studies suggest that when two different types of viruses infect the same cell, their genome segments can mispair and exchange, resulting in the generation of new types viruses (McDonald et al., 2016). This process allows viruses to acquire new pathogenic characteristics and antigenic combinations, facilitating their immune escape and cross-species transmission (Pérez-Losada et al., 2015). In this study, we comprehensively characterized, for the first time, the molecular characters of a naturally occurring reassortant and recombinant cIBDV strain GXYL211225, and evaluated its pathogenicity in Three-yellow chickens, number one breed of Yellow-chicken in southern China.

## 2 Materials and methods

### 2.1 Clinical case, virus isolation, and identification

In December 2021, a poultry farm in Guangxi province experienced suspected IBDV infection in a vaccinated flock of 35-day-old Yellow-chicken. Clinical signs in the affected birds included severe depression, ruffled feathers, and watery or white diarrhea. Necropsy revealed severe hemorrhaging of BF and displayed typical IBD pathological features like muscular hemorrhaging, purple grape shaped BF. Laboratory examinations were conducted on the BF tissue samples collected from the diseased birds. Out of 12 samples detected, a total of 5 samples were found positive (5/12) of IBDV by the RT-PCR detection (Wei et al., 2004). The tissue suspensions of the positive sample were sterile-filtered through a 0.22 μm filter and then used to inoculate SPF chicken embryos for virus isolation as the previous description (He et al., 2012). The isolates, using the tissue suspensions of the infected embryos were identified through the amplification and sequencing analysis of the VP1 and VP2 genes of IBDV, following the method descripted previously (He et al., 2014).

### 2.2 Cell culture, purification, propagation, growth characteristics, and virus titer determination

The isolate was inoculated onto the monolayers of chicken embryo fibroblast (CEF), chicken fibroblast cell line (DF1) and chicken macrophages cell line (HD11) cultured cells, and continually passaged for 3 passages to assess the virus’s growth characteristics, such as cytopathic effects (CPE) and plaque formation (He et al., 2019). Following the method outlined by Wang et al. (2022b), the virus was purified by the plaque technique and then propagated in CEF. Briefly, CEF cells were seeded onto a six-well plate at a density of 5 × 10^5^ cells per well. After 80–85% cells confluent, the medium was removed and the monolayer of cells were infected with 500 μL of each of the 10-fold series virus diluted in DMEM without fetal bovine serum (FBS). After 1 h of virus adsorption at 37°C, the cells were washed twice and then add 2 mL of overlay medium (1 volume of DMEM with 10% FBS and 1 volume of 2% agar) to each well. The overlay medium in the plates were allowed to set for 30 min to solidify, and then inverted the plates and continue incubating at 37°C. The second overlay (containing 0.03% neutral red) was added at 1–2 days post-infection (dpi) and further incubated at 37°C for 24 h to observe the formation of plaques. The titer of the harvested viruses of the purified clone was determined and expressed as 50% tissue culture infectious dose (TCID_50_) (He et al., 2016).

### 2.3 RNA isolation, full-genome amplification, and sequence analysis

Total RNA was isolated from purified viruses using Spin Column Animal Total RNA Purification Kit (Sangon Biotech, China) according to the manufacturer’s instructions and the RNA concentration was determined using a BioDrop ultramicro spectrophotometer (BioDrop, England). Approximately 2 μg RNA was reverse-transcribed into cDNA for each sample using the All-In-One 5X RT MasterMix Kit (abm, Canada) according to the manufacturer’s protocol. Using the obtained cDNA as a template, PCR amplification of the full-genome was carried out with primers as described in previously study (Wang et al., 2022b). The PCR products were purified by gel recovery and ligated into the pMD18-T vector, and then transformed into the *E. coli* DH5α competent cells. At least three individual positive clones of each fragment were selected for sequencing (BGI, Guangzhou, China). Analysis of the nt homology and amino acid (aa) alignment for both segment A and B of the isolate were conducted using MEGA 6.0 software. Furthermore, maximum likelihood (ML) phylogenetic trees were constructed using the MEGA 6.0 program with the best-fit model (GTR + I + G). The full-genome of the segments A and B of the isolate (named GXYL211225) were submitted to GenBank (accession No. OR523680 and OR523681).

### 2.4 Genome recombination analysis

Two different recombination detection methods were used to identify the potential recombination events in the full-genome of GXYL211225 as the previous report (Wang et al., 2020a, 2022a). Initially, both the two datasets, A and B, were tested by RDP software, which is regarded as reliable when at least four out of seven distinct algorithms (RDP, GENECONV, Bootscan, MaxChi, Chimera, SiScan, and 3Seq) detect significant recombination signals (*p* < 10E-6). Subsequently, potential recombination events were further validated using similarity plots in Simplot.

### 2.5 Pathogenesis experiments

A total of 40 1-day-old non-vaccinated Three-yellow chickens were purchased from a local commercial poultry farm. The birds were randomly divided into 4 groups (Groups 1, 2, 3, 4; n = 10) and housed in separate isolators, where they had unrestricted access to water and feed. At 28 days, serum samples were collected from all birds for ELISA to determine the antibody levels and ensure the exclusion of the maternal antibody interference during the subsequent infection (Wang et al., 2020b). The birds of Group 1, 2, and 3 were orally administered with 10^5^ TCID_50_/0.5 mL viruses of vvIBDV strain NN1172 (genotype A2B3) (He et al., 2014), nvIBDV strain QZ191102 (genotype A2dB1b) (Wang et al., 2022b) and the isolate GXYL211225 (genotype A1aB1a) (cIBDV strain), respectively, while Group 4 received 0.5 mL of PBS buffer. Daily clinical symptom observations were recorded, and clinical symptom were quantified by the mean symptom index (MSI) score (Nouen et al., 2012), which is based on four degrees: 0 = “lack of signs,” 1 = “typical IBD signs (ruffled feathers) conspicuous in quiet bird only, the bird stimulated by a sudden change in environment (light, noise, or vicinity of experiment observer) appears normal, motility is not reduced”; 2 = “typical IBD signs conspicuous even when bird is stimulated, dehydration is apparent, motility may be slightly reduced” and 3 = “typical severe IBD signs with prostration or death.” At 7 days post-challenge (dpc), all birds were euthanized and necropsied. The BF was taken and weighed, and the bursa/body weight ratio index (BBIX) was calculated, a BBIX value of less than 0.7 was considered as atrophy. Half of the bursa tissue from the birds was fixed in 4% neutral buffered formaldehyde for histopathological examination and quantified according to Skeeles’ scale for histopathological bursal lesion scores (Skeeles et al., 1979), which is based on five degrees: 0 = no lesions; 1 = mild scattered cell depletion in a few follicles; 2 = moderate, one-third to one half of the follicles have atrophy or depletion of cells; 3 = diffuse, atrophy of all follicles; and 4 = acute inflammation and acute necrosis typical of IBD. While the other half of the bursa tissue was used for RNA extraction.

### 2.6 Quantitative RT-PCR assay targeting the IBDV

qRT-PCR assay targeting the IBDV was conducted on the extracted RNA samples using the previously descripted method (He et al., 2016). Briefly, the qRT-PCR reaction was conduct with the primer IBDV-F 5′-GGAGCCTTCTGATGCCACA-3′ and IBDV-R 5′-ATTGTAGGTCGAGGTCTCTG-3′. Firstly, total RNA was isolated from each sample using Spin Column Animal Total RNA Purification Kit (Sangon Biotech, China) according to the manufacturer’s instructions and the concentration was determined using a BioDrop ultramicro spectrophotometer (BioDrop, England). Secondly, approximately 2 μg RNA was reverse-transcribed into cDNA for each sample using the All-In-One 5X RT MasterMix Kit (abm, Canada) according to the manufacturer’s protocol. Finally, the qRT-PCR analysis was performed in a reaction volume of 20 μL containing 10 μL of 2 × ChamQ Universal SYBR qPCR Master Mix, 0.4 μL of each primer, 7.2 μL of ddH_2_O and 2 μL of cDNA. Thermal cycling was performed by using two-step real-time PCR at 95°C for 30 s, followed by 40 cycles of 95°C for 10 s and 55°C for 30 s. Fluorescence values were read and recorded for RT-PCR analysis.

### 2.7 Statistical analysis

All data in this study were processed using GraphPad Prism 8.0 software and presented as mean ± standard deviation (SD). Statistical differences among the groups were evaluated using one-way analysis of variance (ANOVA). To compare the differences among treatments, Tukey’s multiple comparisons test was used. Results with *p* values of < 0.05 *, *p* < 0.01 **, *p* < 0.001 ***, *p* < 0.0001 **** were considered statistically significant.

## 3 Results

### 3.1 Isolation and identification of a cIBDV strain from clinical case

**Figure 1:**
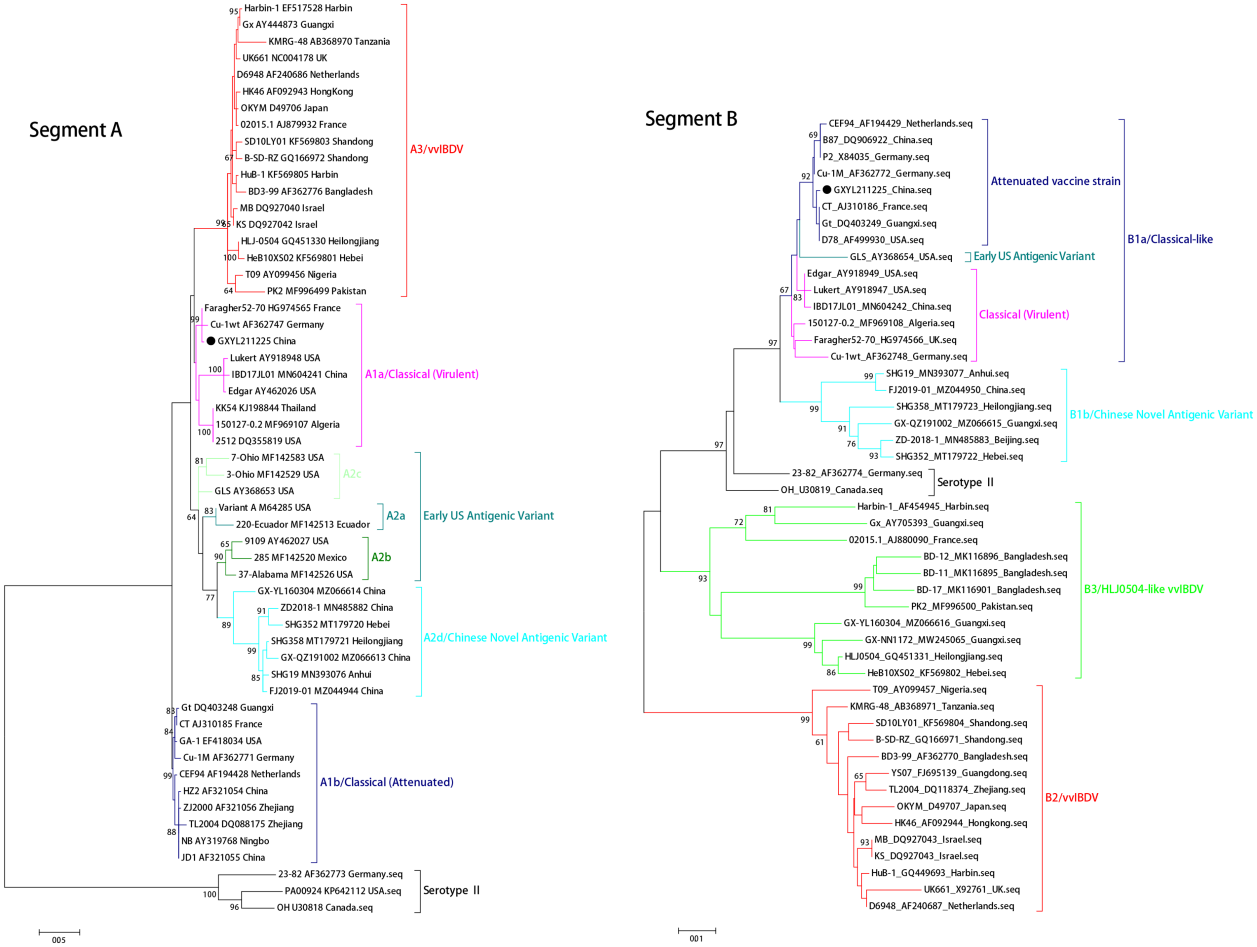
Maximum likelihood tree (ML tree) was constructed using the datasets of 55 *v*VP2 sequence (474 bp, nt 737–1,210) and 48 VP1 sequence (767 bp, nt 756–1,522) by the MEGA 6.0 software with the best-fit model (GTR + I + G) with 1,000 ultrafast bootstrap replicates. The strain GXYL211225 was labeled with ● mark in the figure.

The isolate demonstrated the capability to induce mortality in SPF chicken embryos as early as the first passage, showing severe subcutaneous hemorrhaging within 36 h post-inoculation. Amplification and sequencing analysis of the VP1 and VP2 genes of the isolate confirmed that the isolated strain in the clinical samples belonged to cIBDV (Figure 1) and was named GXYL211225.

### 3.2 GXYL211225 was further confirmed to be a natural reassortant strain by the full-length genome sequencing and analysis

**Figure 2:**
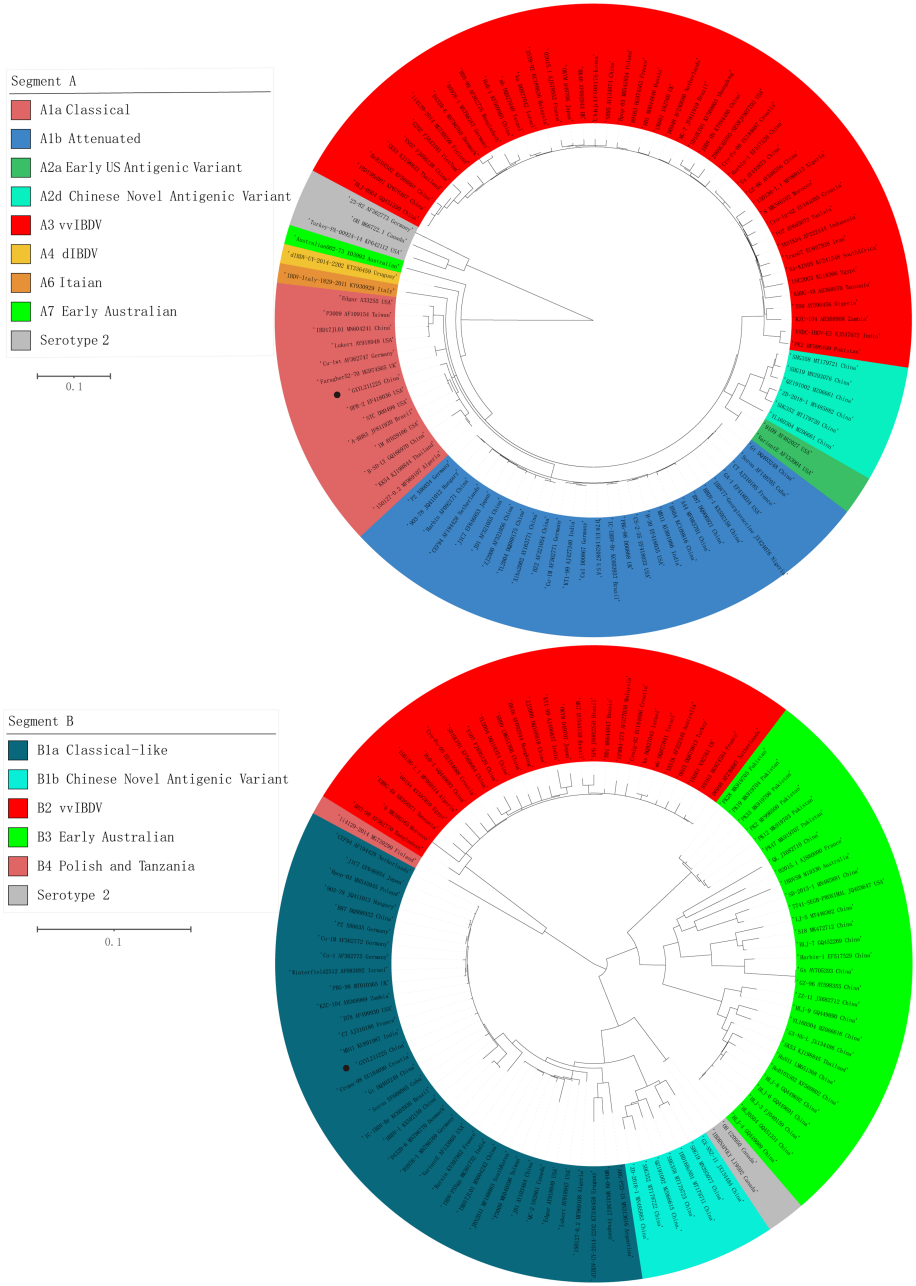
Maximum likelihood tree (ML tree) was constructed using the datasets of 102 strains of IBDV with both the full-length segments A (3,260 bp) and B (2,827 bp) by the MEGA 6.0 software with the best-fit model (GTR + I + G) with 1,000 ultrafast bootstrap replicates. The strain GXYL211225 was classified as A1aB1a and was labeled with ● mark in the figure.

The ML phylogenetic tree of the full-length segments A and B of GXYL211225 were shown in Figure 2, providing further evidence that this isolated is a natural reassortant strain (A1aB1a) according to the newly proposed unified classification and nomenclature system (Jackwood et al., 2018; Islam et al., 2021; Wang Y. L. et al., 2021; Wang et al., 2022b). The phylogenetic tree of the segment A revealed that GXYL211225 clustered with the classical virulent strains (A1a) like Faragher52-70, 150127–0.2 and STC, demonstrating the typical characteristics of the A1a classical strains. Notably, key aa residues associated with cIBDV were observed in the VP5 protein, where critical sites 3G, 45G, 74I, 87G, 125S matched those of the classical strain 150127–0.2 originating from Algeria (Table 1). Similar cases can be observed in the polyproteins VP2-VP4-VP3, with the exception of seven unique aa residues mutations 10R, 76G, 142S, 242I, 294 L, 802 V, and 942 T, all the characteristic aa residues in VP2-VP4-VP3 were also completely consistent with the classical strain 150127–0.2 (Table 1).

**Table 1:** The differences of the aa residues among the cIBDV, attIBDV, vvIBDV, early US avIBDV and nvIBDV isolates in VP5, polyprotein and VP1.

		Segment A																																			Segment B													
		VP5					Polyprotein (VP2-VP4-VP3)																														VP1													
Strain	Phenotype	3	45	74	87	125	10	76	142	213	222	242	249	253	254	256	279	284	286	294	299	318	451	453	521	541	680	685	686	715	751	802	923	942	981	1,005	4	13	61	145	146	147	251	287	492	508	511	546	646	687
GXYL20211225	Reassortment	G	G	I	G	S	R	G	S	D	P	I	Q	Q	G	V	D	A	T	L	N	G	I	K	T	V	C	K	V	P	H	V	K	T	P	T	I	T	V	N	E	G	R	T	D	R	R	L	G	S
150127-0.2	c	*	*	*	*	*	Q	S	N	*	*	V	*	*	*	*	*	*	*	I	*	*	*	*	*	*	*	*	*	*	*	A	*	I	*	*	*	K	*	*	*	D	K	*	N	*	*	P	*	*
Faragher52-70	c	*	*	*	E	P	Q	S	N	*	*	*	*	*	*	*	*	*	*	*	*	*	*	R	A	*	*	*	*	*	*	A	R	I	*	*	*	K	*	*	*	*	K	*	N	*	*	P	*	*
IBD17JL01	c	S	*	*	E	P	Q	S	N	*	L	*	H	*	*	A	*	T	I	*	*	*	*	R	A	*	*	*	*	*	*	A	R	I	*	*	*	K	*	*	*	*	K	*	N	*	*	P	*	*
Cu-1wt	c	S	*	*	E	P	Q	S	N	*	*	*	*	*	*	*	*	*	*	*	*	*	*	R	A	*	*	*	*	*	*	A	R	I	*	*	*	K	*	*	*	*	K	*	N	*	*	P	*	*
B87	att	S	*	*	*	*	Q	*	N	*	*	V	*	H	*	*	N	T	*	*	*	*	*	R	A	*	*	*	*	*	*	A	R	I	L	*	*	*	*	*	*	*	K	*	N	*	*	*	*	*
Gt	att	S	*	*	*	*	Q	*	N	*	*	V	R	H	*	*	N	T	*	*	*	*	*	R	A	*	*	*	*	*	*	A	R	I	L	*	*	*	*	*	*	*	K	*	N	*	*	*	*	*
P2	att	S	*	*	E	*	Q	S	N	*	*	V	R	H	*	*	N	T	*	*	*	*	*	R	A	*	*	*	*	*	*	A	R	I	L	*	*	*	*	*	*	*	K	*	N	*	*	*	*	*
CT	att	S	*	*	*	*	Q	*	N	*	*	V	R	H	*	*	N	T	*	*	*	*	*	R	A	*	*	*	*	*	*	A	R	I	L	*	*	*	*	*	*	*	K	*	N	*	*	*	*	*
UK661	vv	S	R	L	E	P	Q	S	N	*	A	*	*	*	*	I	*	*	*	I	S	*	L	R	A	I	Y	N	*	S	D	A	R	I	*	A	V	K	I	T	D	N	K	A	N	K	S	P	S	P
HK46	vv	S	R	F	E	P	Q	S	N	*	A	*	*	*	*	I	*	*	*	I	S	*	L	R	A	I	Y	N	*	S	D	A	R	I	*	A	V	K	I	T	D	N	K	A	N	K	S	P	S	P
HLJ-0504	vv	S	R	F	E	P	Q	S	N	*	A	*	*	*	*	I	*	*	*	I	S	*	L	R	A	I	Y	N	*	S	D	A	R	I	*	A	V	K	I	T	*	*	K	A	N	K	S	P	S	P
NN1172	vv	S	R	F	E	P	Q	S	N	*	A	*	*	*	*	I	N	*	*	I	S	*	L	R	A	I	Y	N	*	S	D	A	R	I	*	A	V	K	I	T	*	*	K	A	N	K	S	P	S	P
Variant_E	av	S	*	*	E	P	Q	S	N	N	T	V	K	*	S	*	N	*	I	*	*	D	*	R	A	I	*	*	I	*	*	A	R	I	*	*	*	K	*	*	*	*	K	*	N	*	*	P	*	*
9109	av	S	*	*	E	P	Q	S	N	N	T	V	K	*	N	*	N	*	I	*	*	N	*	R	A	I	Y	*	I	*	*	A	R	I	Q	A	*	*	*	*	*	D	K	*	N	*	*	*	*	*
QZ191002	nv	R	*	L	E	P	Q	S	N	N	T	V	K	*	N	*	N	*	I	*	S	D	L	R	A	I	*	*	I	*	*	A	R	I	*	A	*	K	*	*	*	D	K	*	N	K	*	P	*	*
SHG19	nv	R	*	L	E	P	Q	S	N	N	T	V	K	*	N	*	N	*	I	*	S	D	L	R	A	I	*	*	I	*	*	A	R	I	*	A	*	K	*	*	*	D	K	*	N	K	*	P	*	*

The phylogenetic tree of segment B sequence showed that GXYL211225 clustered with the classical-like (B1a) attenuated vaccine strain (Figure 2). The segment B exhibited close similarity to the commonly used attenuated vaccine strains in China, such as B87 and CT, sharing all characteristic aa residues 4I, 13 T, 61 V, 145 N, 146E, 147G, 287 T, 508R, 511R, 546 L, 646G, and 687S, except for two unique aa residues 251R and 492D (Table 1). Comprehensive analysis of the full-genome segments A and B suggested that GXYL211225 is a naturally occurring reassortant strain, which segment A originates from the classical strains (A1a) and segment B originates from the attenuated vaccine strains (B1a).

### 3.3 Wide cell tropism and prominent cytopathic effects in different cells

**Figure 3:**
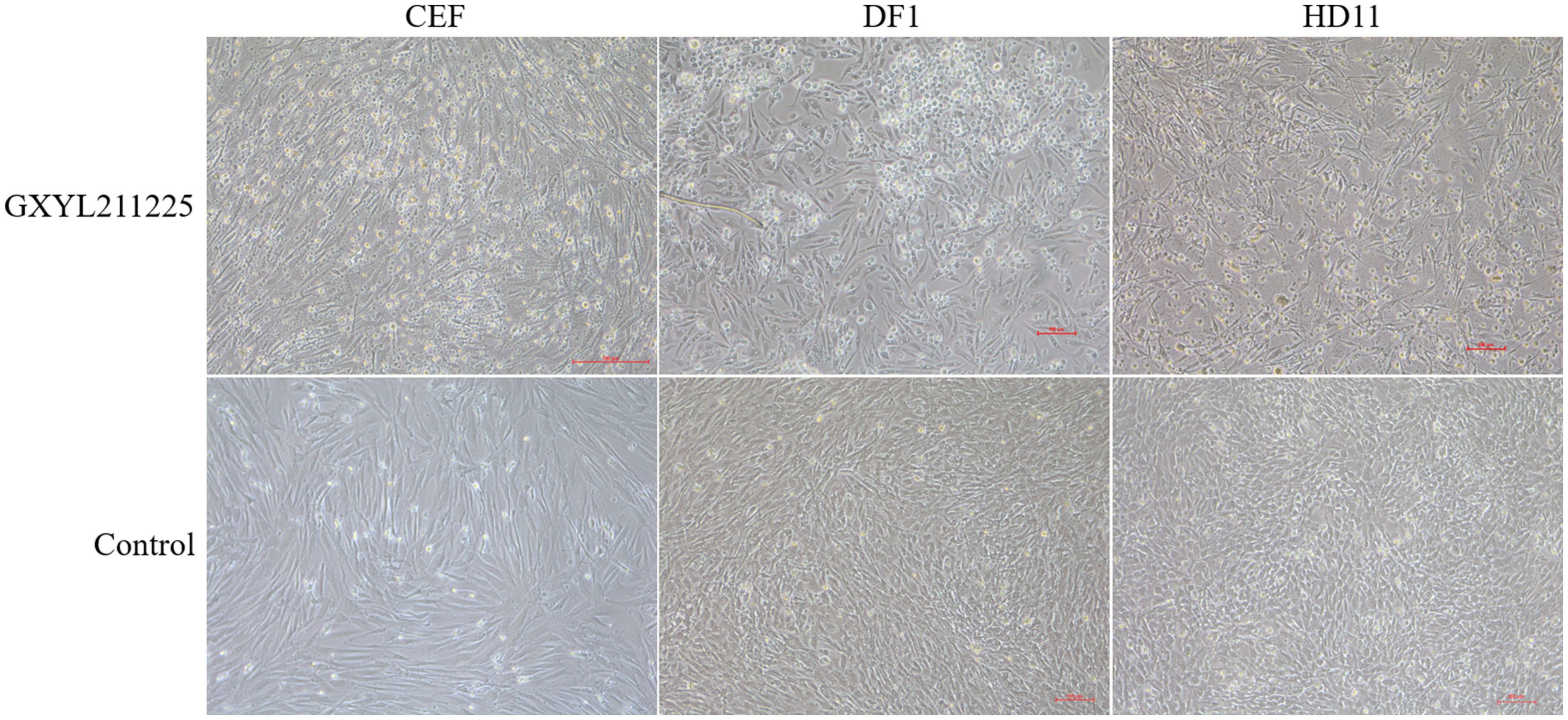
The CPEs of CEF, DF1, and HD-11 monolayer cells inoculated with the isolate GXYL211225 at 36 h post-infection.

The CPEs induced by the GXYL211225 strain on the commonly used cell lines CEF, DF1, and HD11 were depicted in Figure 3. GXYL211225 inoculated on the CEF, DF1, and HD11 monolayer cells all grown well after three blind passages, and prominent CPEs were evident as early as 36 h post-inoculation. The hallmark characteristics of CPEs included rounding, enhanced refractive index, cell rupture, and the detaching of the cells from the monolayer. As expected, the cells in the control group growth normally and no CPE was observed. It is noteworthy that significant CPEs were observed in the first passage of CEF monolayer infected with the isolate, and this pronounced effect persisted through the subsequent passages.

### 3.4 Detection of significant recombination events in segment A of GXYL211225

**Figure 4:**
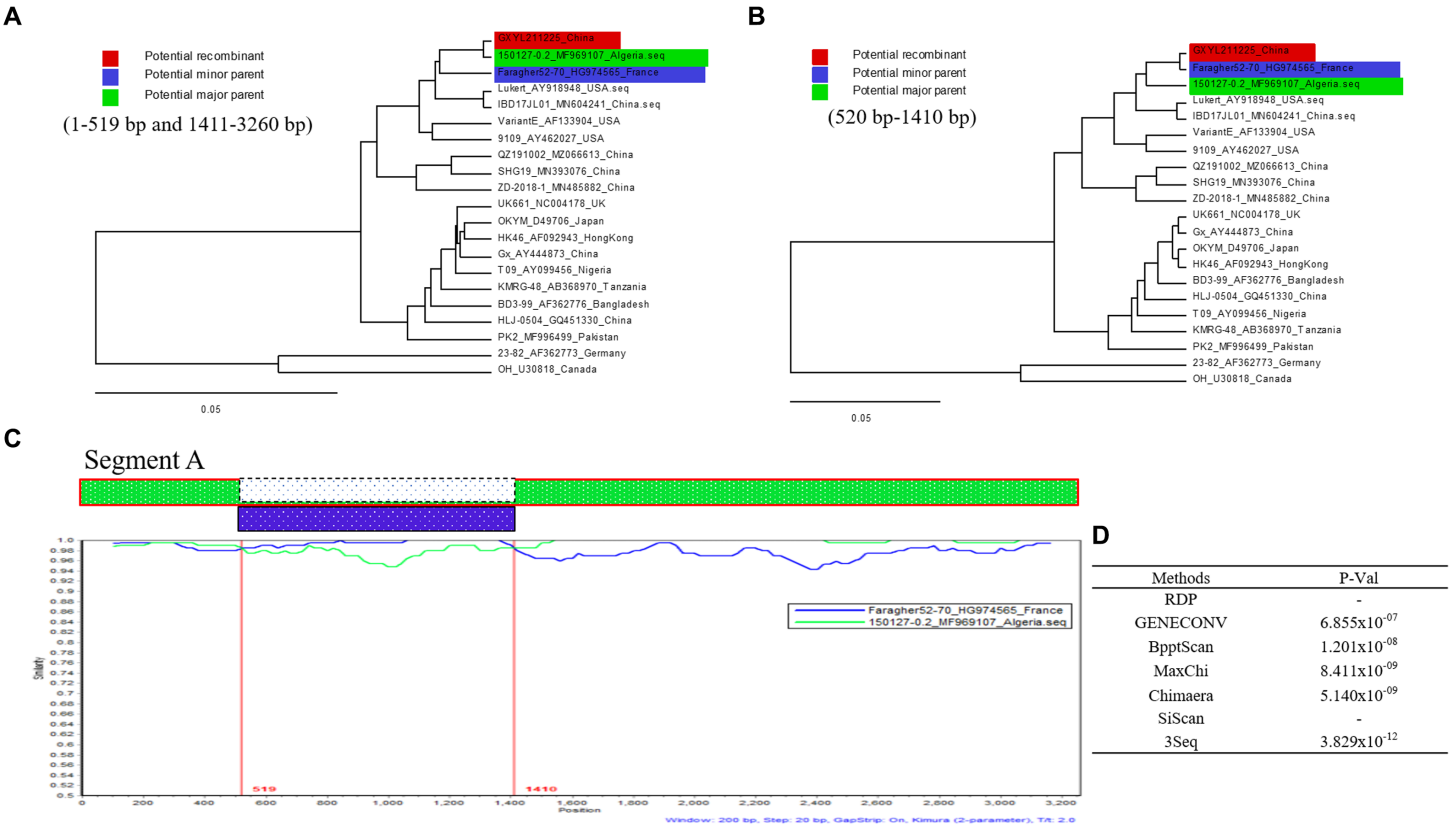
Recombination analysis of the GXYL211225 strain. **(A,B)** Maximum-likelihood phylogenetic profiles of separate regions in the segment A of reference strain (of 21 strains, 3,260 bp). Panel **(A)** represents the 5′ region (nt 1–519) and 3′ region (nt 1,411–3,260) sections of segment A. Panel **(B)** represents the region (nt 520–1,410) section of segment A; **(C)** Comparison of the similarity plots of segment A scales (query) with 150127-0.2 (green) and Faragher52-70 (blue); **(D)** The P-Val of seven different algorithms.

Five out of the seven different algorithms (Figure 4D) detected a significant recombination event (*p* < 10E-6) within the segment A of GXYL211225. Segment A is a result of recombination between the classical strain 150127–0.2 (major parent) and Faragher52-70 (minor parent) (Figures 4A,B). The recombination breakpoints were identified at the position of nt 519–1,410, and manifested as a backbone originating from the classical strain 150127–0.2, wherein a fragment located at the position of nt 519–1410 is replaced by the corresponding part from Faragher52-70 strain (Figure 4C).

### 3.5 Naturally occurring reassortant and homologous recombinant strain GXYL211225 cause mortality and immune organ damages in three-yellow chickens

**Figure 5:**
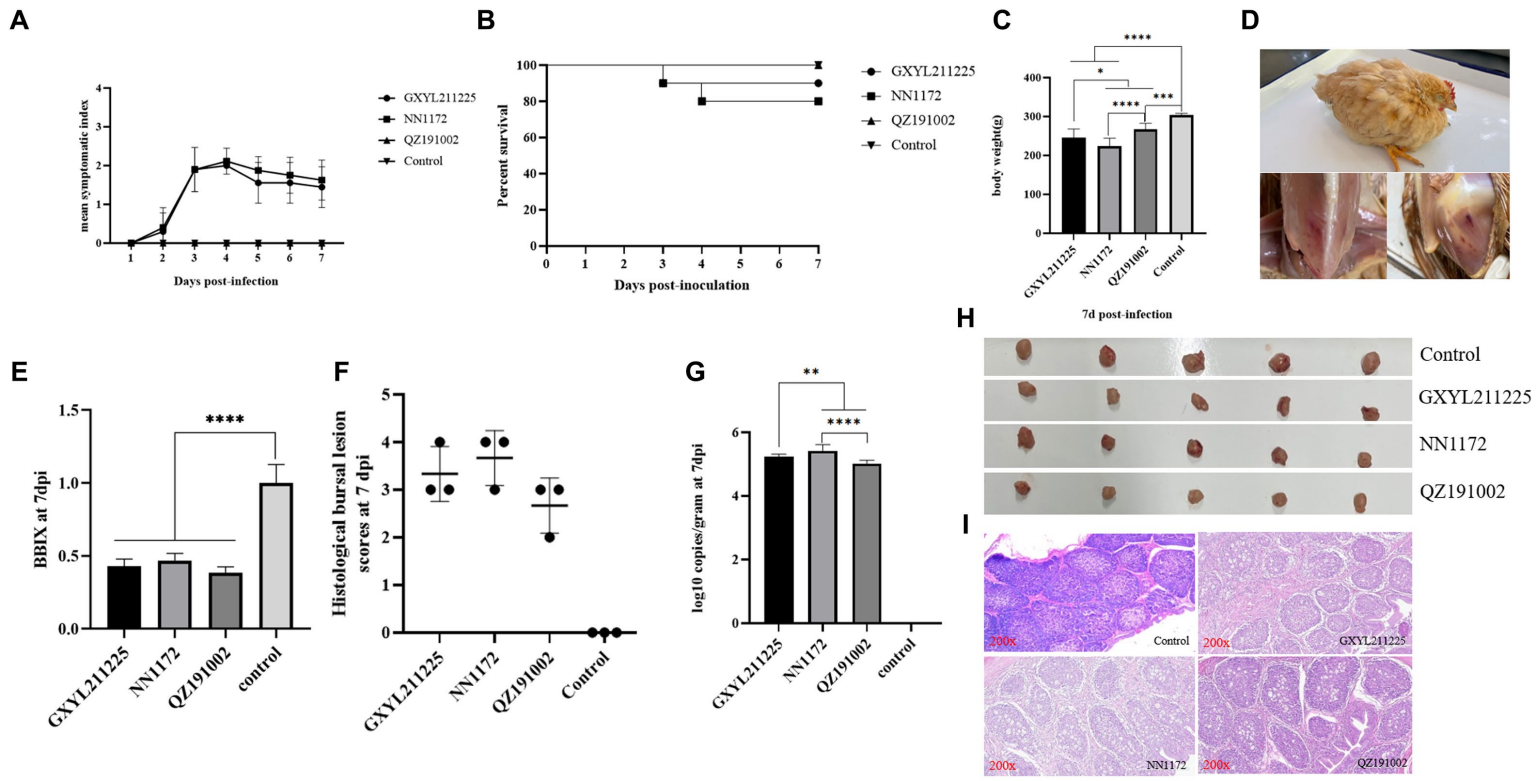
Pathogenic analysis of GXYL211225, QZ191002, and NN1172 strains in Three-yellow chickens. **(A)** Mean symptom index (MSI) scores; **(B)** Survival rates; **(C)** Body weights; **(D)** Typical clinical symptoms and pathological changes challenged with GXYL211225; **(E)** The bursa/body weight index (BBIX) ratio; **(F)** Histopathological bursal lesion scores (HBLS); **(G)** Viral loads of IBDV in the BF tissues; **(H)** The BF images at 7 dpc; **(I)** Histopathological changes of BF at 7 dpc (200×). Results with *p* values of < 0.05 *, *p* <  0.01 **, *p* <  0.001 ***, *p* <  0.0001 **** were considered significant different.

The pathogenicity of GXYL211225 in Three-yellow chickens was assessed and compared to the HLJ-0504-like vvIBDV strain NN1172 and the nvIBDV strain QZ191002 (Figure 5). All serum samples tested negative by ELISA, indicating that the maternal antibody has been decreased to a very low level at 28d. During the whole experiment, no obvious clinical symptoms or mortality was observed in the control group and the QZ191002 group (Figure 5A). In the NN1172 and GXYL211225 groups, typical IBD symptoms such as disheveled feathers, depression, diarrhea were observed, with mortality rates of 20% (2/10) and 10% (1/10), respectively (Figures 5B,D). Compared to the control group, birds in all the challenged groups showed significant body-weight loss (Figure 5C). Upon necropsy, bursal atrophy in all the challenged groups (Figures 5E,H), and typical hemorrhages in pectoral and thigh muscle were observed (Figure 5D). Histopathological examination of bursa sections revealed the most severe lesions happened in the NN1172 group, characterized by loss of follicular structural contour and replacement by the proliferated connective tissue and reticular cells; followed by the GXYL211225 group, showing moderate to severe lymphocyte necrosis and loss of follicular structural contours; and then the QZ191002 group with moderate lesions (Figures 5F,H,I). Viral loads (virus copy numbers) detected in the BF tissue were consistent with the histopathological changes (Figures 5F,G). These data taken together confirm that the naturally occurring reassortant and recombinant strain GXYL211225 exhibited lower pathogenicity in chickens when compared to vvIBDV, and higher pathogenicity when compared to nvIBDV.

## 4 Discussion

In China, the infection caused by cIBDV strain was first reported in 1979, resulting in significant economic losses to the poultry industry at that time. However, with the emergence of vvIBDV in 1991 (Zhang et al., 2022), there has been an increasing focus on these strains in China, while reports related to cIBDV have been scarce. The research conducted by our group revealed that since 2020, classical strains have gradually re-emerged in southern China and have undergone different types of reassortment events with locally prevalent strains (unpublished data). Subsequently, classical strains were also reported in Hubei province of central China (Lian et al., 2022). These classical strains have caused significant economic losses due to their widespread circulation in the field. Furthermore, the use of attenuated live vaccines has substantially increased the likelihood of reassortment and recombination events involving the classical strains. Therefore, considering the re-emergence of classical strains and their potential to undergo reassortment and recombination with locally circulating strains, a continuous and in-depth understanding of the genetic variation and pathogenic characteristics of this type of strains is of paramount importance for better control of the disease in the poultry industry.

Reassortment is a crucial mechanism in the evolution of viruses, contributing to the generation of new strains in viruses with segmented genomes (Hon et al., 2008; He et al., 2009; Pérez-Losada et al., 2015; McDonald et al., 2016; Feng et al., 2022; Wang et al., 2022a,b). Studies confirmed that isolate GXYL211225 was a naturally occurring reassortant and recombinant strain of cIBDV. Its segment A belongs to the classical strain (A1a), while segment B derives from the classical-like (B1a) attenuated vaccine strain (Figure 2). Further analysis of the recombination in its segment A reveals a significant recombination event was found. Specifically, segment A is a result of recombination between the classical strain 150127–0.2 (major parent) and Faragher52-70 (minor parent), characterized by a backbone originating from the classical strain 150127–0.2 (Abed et al., 2018), where a fragment located at nt 519–1410 is replaced by the corresponding part of the Faragher52-70 strain (Figure 4). Two factors can explain this case: firstly, the gradual prevalence of strains like Faragher52-70 in southern China since 2020, including isolates such as NN200430, NN200519, and YL200617 etc., have provided opportunities for the recombination; Secondly, it’s possible that the 150127–0.2 strain, belonging to cIBDV, may have altered its antigenicity after recombination with Faragher52-70 strain at nt 519–1410, resulting from the mutations of the critical antigenic activity residues V242I and I294L, thereby escaped the immune protections from the cIBDV vaccines, and also the widely used vvIBDV vaccines (resulting from the mutations of the critical antigenic activity residues A222P, I256V, I294L, and S299N) over the past two decades (Table 1). Additionally, the segment B of the GXYL211225 isolate originated from the attenuated vaccine strain, exhibiting a high homology with the commonly used vaccine strains in China (Figure 1). Considering that the isolate GXYL211225 was obtained from a vaccinated flock, it is speculated that this strain may have resulted from the reassortment between the re-emerging classical strains, observed in recent years, with the widely used attenuated vaccine strains in clinical practice. The identification of this strain indicated that classical strains are undergoing rapid evolution through multiple mechanisms, including not only the common site mutations but also the simultaneous processes of genetic reassortment and homologous recombination. The emergence of these novel-featured strains may contribute to their ability to escape the protection offered by current vaccines, and further immunization-challenge experiments will be necessary to validate this in the future.

The diversity-driven evolution of viruses contributes to changes in aspects such as pathogenicity and cell tropism, allowing newly emerging viruses to adapt to the new hosts and environments for better survival (Pérez-Losada et al., 2015; McDonald et al., 2016). The study revealed that GXYL211225 was able to cause death in all SPF chicken embryos within 36 h post-inoculation even just in the first passage, with severe subcutaneous hemorrhage lesion. Compared to the previous reported strains that required 2–3 continuous passages on chicken embryos to induce significant lesions and death (He et al., 2016; Chen et al., 2018), the ability of GXYL211225 to cause embryos pathogenic lesions and death without the need of adaptation suggests the presence of new and uncharacterized characters. Previous studies have confirmed that the crucial aa residues 253H, 279 N and 284A in VP2 are associated with IBDV’s cell tropism (Brandt et al., 2001; Qi et al., 2009; Ben Abdeljelil et al., 2014; Noor et al., 2014), considered as determining factors for the cell tropism. Notably, GXYL211225 exhibited mutations H253Q, N279D, and A284T at these crucial cell tropism-related aa residues in VP2, yet still displayed wide cell tropism (Figure 3). This suggests the presence of other crucial aa residues impacting IBDV’s cell tropism that have yet not to be full characterized. Increasing evidence indicates that IBDV’s cell tropism is a result of interactions among multiple genes or crucial aa residues within its genome. Besides 253H, 284A, and 279 N in VP2, aa residues like 222P and 256 V have also been implicated in IBDV’s cell tropism (Qi et al., 2013, 2016), which is consistent with the characteristics of GXYL211225. Additionally, mutations at critical aa residues Y680C, N685K, S715P, D751H, V990A, and A1005T in VP4 and VP3 proteins of GXYL211225 also contribute to the virus adaptation to cells. This is consistent with the previous reports of aa mutations associated with cell adaptation to the attenuation Gt strain obtained by continuous passage in the embryos and cells from vvIBDV GX strain (Wang et al., 2007, 2010). Furthermore, VP5 protein has also been found to impact the replication efficiency and pathogenicity of IBDV *in vitro* (Qin et al., 2009, 2010). Recent studies suggest that VP1 protein contributes to IBDV’s replication and virulence (Boot et al., 2005; Escaffre et al., 2013; Gao et al., 2018), with mutations like V4I and P687S enhancing the replication capacity *in vitro* (Yu, 2011; Yu et al., 2013; Wang W. et al., 2021). Factors associated with cell tropism remain intricate, and the extensive cell tropism exhibited by this newly emerged recombinant strain requires further investigation in the future.

Pathogenicity experiments confirmed that GXYL211225 exhibited stronger pathogenicity in the SPF chicken embryos and the commercial Three-yellow chickens compared with the nvIBDV strain QZ191002. Throughout the experiment period, the birds in QZ191002 challenged group and the control group showed no obvious clinical symptoms, as the previous reports (Fan et al., 2019; Chen et al., 2022; Wang et al., 2022b). In contrast, in the vvIBDV strain NN1172 and GXYL211225 challenged groups, birds exhibited mortality rates of 20% (2/10) and 10% (1/10), respectively (Figure 5). Infection of GXYL211225 resulted in similar symptoms as those of NN1172, characterized by ruffled feathers, watery white feces, reduced weight, hemorrhage in pectoral and thigh muscle, and BF atrophy (BBIX<0.7). Previous studies have shown that the virulence can also change when IBDV undergoes reassortant and/or recombination occurs in the field (Hon et al., 2008; He et al., 2009; Jackwood et al., 2011; Lu et al., 2015; Wu et al., 2020; Feng et al., 2022; Nooruzzaman et al., 2022; Wang et al., 2022b). Compared to the earlier cIBDV strain Faragher52-70 as previously reported (Bulow et al., 1985), the isolated GXYL211225 exhibited a lower mortality rate and significantly less BF damage. This may be attributed to the fact that the B-segment of this isolated strain is derived from an attenuated vaccine strain (He et al., 2016; Wang W. et al., 2021). With the emergence of GXYL211225 and the strains of similar recombination and reassortment, this type of strains may have the potential to escape the protections of the currently used vaccines (vvIBDV or/and attIBDV), posing a significant threat to the industry in the future.

In conclusion, this study isolated and characterized a naturally occurring classical IBDV strain GXYL2111225 (genotype A1aB1a) with unique recombination and reassortment events for the first time. This classical IBDV strain with distinct molecular characteristics exhibits extensive cell-tropism. Its virulence was intermediate between those of the vvIBDV strain NN1172 and the nvIBDV strain QZ191002, causing a 10% mortality rate and severe immune organ damages in the commercial chickens. Furthermore, our research findings confirmed the rapid and diverse evolution of the re-emerged classical strains in China and emphasizes the need to monitor and characterize the evolution of IBDV in the field, including in the aspects of both genetics and pathogenicity, for the effective control of the disease.

## Data availability statement

The original contributions presented in the study are publicly available. This data can be found at: https://www.ncbi.nlm.nih.gov/; OR523680-OR523681.

## Ethics statement

Animal experiments in this study were approved by the Animal Experimental Ethical Committee of Guangxi University (GXU-2022-314). The study was conducted in accordance with the local legislation and institutional requirements.

## Author contributions

WW: Data curation, Writing – original draft. JS: Data curation, Writing – original draft. YZ: Investigation, Writing – original draft. YQ: Investigation, Writing – original draft. WZ: Formal analysis, Writing – original draft. ZW: Formal analysis, Writing – original draft. TN: Formal analysis, Writing – original draft. SH: Formal analysis, Writing – original draft. YC: Formal analysis, Writing – original draft. RK: Formal analysis, Writing – original draft. XH: Writing – review & editing. PW: Conceptualization, Funding acquisition, Methodology, Writing – review & editing.
